# Trajectories of vital status parameters and risk of mortality among acute organophosphorus poisoning patients – a latent class growth analysis

**DOI:** 10.1186/s12889-020-09637-x

**Published:** 2020-10-12

**Authors:** Waqas Ahmed Farooqui, Mudassir Uddin, Rashid Qadeer, Kashif Shafique

**Affiliations:** 1 Department of Statistics, University of Karachi, Karachi, Pakistan; 2 School of Public Health, Dow University of Health Sciences, Karachi, Pakistan; 3 Department of Medicine, Dr. Ruth K.M. Pfau/Civil Hospital Karachi, Dow University of Health Sciences, Karachi, Pakistan; 4 Institute of Health and Wellbeing, University of Glasgow, Glasgow, UK

**Keywords:** Latent growth curve, OP poisoning, Vital signs, Repeated measures

## Abstract

**Background:**

Acute organophosphorus (OP) poisoning is one of the major causes of mortality among patients presenting to emergency departments in developing countries. Although various predictors of mortality among OP poisoning patients have been identified, the role of repeated measurements of vital signs in determining the risk of mortality is not yet clear.

Therefore, the present study examined the relationship between trajectories of vital signs and mortality among OP poisoning patients using latent class growth analysis (LCGA).

**Methods:**

This was a retrospective cohort study using data for 449 OP poisoning patients admitted to Civil-Hospital Karachi from Aug’10 to Sep’16. Demographic data and vital signs, including body temperature, blood pressure, heart rate, respiratory rate, and partial-oxygen pressure, were retrieved from medical records. The trajectories of vital signs were formed using LCGA, and these trajectories were applied as independent variables to determine the risk of mortality using Cox-proportional hazards models. *P*-values of < 0.05 were considered statistically significant.

**Results:**

Data for 449 patients, with a mean age of 25.4 years (range 13–85 years), were included. Overall mortality was 13.4%(*n* = 60). In trajectory analysis, a low-declining systolic blood pressure, high-declining heart rate trajectory, high-remitting respiratory rate trajectory and normal-remitting partial-oxygen pressure trajectory resulted in the greatest mortality, i.e. 38.9,40.0,50.0, and 60.0%, respectively, compared with other trajectories of the same parameters. Based on multivariable analysis, patients with low-declining systolic blood pressure were three times [HR:3.0,95%CI:1.2–7.1] more likely to die compared with those who had a normal-stable systolic blood pressure. Moreover, patients with a high-declining heart rate were three times [HR:3.0,95%CI:1.5–6.2] more likely to die compared with those who had a high-stable heart rate. Patients with a high-remitting respiratory rate were six times [HR:5.7,95%CI:1.3–23.8] more likely to die than those with a high-stable respiratory rate. Patients with normal-remitting partial oxygen pressure were five times [HR:4.7,95%CI:1.4–15.1] more likely to die compared with those who had a normal-stable partial-oxygen pressure.

**Conclusion:**

The trajectories of systolic blood pressure, heart rate, respiratory rate and partial-oxygen pressure were significantly associated with an increased risk of mortality among OP poisoning patients.

## Background

Acute organophosphorus (OP) poisoning is a global public health challenge. Acute OP poisoning cases have high morbidity and mortality, constituting a challenge for hospital practitioners [1, 2]. A systematic review including data from 102 countries showed that the proportion of suicides due to pesticide self-poisoning varies considerably between regions, from 0.9% in low- and middle-income countries in the European region to 48.3% in low- and middle-income countries in the Western Pacific region [3]. In neighbouring countries, the prevalence of OP poisoning among other types of poisoning varies from 7.7 to 20.7% (20.7% in China [4], 7.7% in Iran [5], and 20.7% in India [6]). Among OP poisoning cases, the case fatality rate varies from 10 to 30% in developing countries [7, 8]. In our urban city, the prevalence of OP poisoning among all poisoning cases is reported to be nearly 46.1%, with a mortality rate of 2.7% [9].

Mortality among patients with acute OP poisoning is considerably high but varies between regions and depends on the quality of healthcare services available to patients. Several other factors, including age, sex, type of poison ingested and its biochemical properties, quantity of poison, time since ingestion, any pre-existing comorbidities and access to health services, influence the outcome of OP poisoning patients [10–14]. The prognosis of acute poisoning depends on the exposure to the toxin as well as the amount of toxin ingested and the physiology of compensation. In our country, it is difficult to judge the amount because patients ingest different brands, and there is a lack of description of the concentration of the poisonous substance [9, 15]. In clinical settings, the prognosis of these patients is mainly assessed by vital status, including body temperature, blood pressure, heart rate and respiratory rate, levels of anticholinesterases, oxygenation and PaCO_2_ for hypoventilation requiring intubation/ventilation. Vital signs play an important role in the diagnosis of intoxicated patients since they are the key components of toxic syndrome. However, their role in assessing the severity of poisoning and prognosis of these patients remains unclear [16].

One-third of all OP poisoning patients also require mechanical ventilation (MV) due to heavy ingestion, the toxicity of the poison ingested and delayed presentation at the hospital [17–19], and among OP poisoning patients on MV, low pseudocholinesterase (PChE), high creatinine (Cr), low Glasgow Coma Scale (GCS) score and long hospitalization durations are all linked with high mortality [10–12, 17].

Many scoring systems are currently being used to evaluate the severity of acute OP poisoning, such as the Acute Physiology and Chronic Health Evaluation (APACHE) II score [20, 21], GCS, Peradeniya Organophosphorus Poisoning Scale (POPS) [22], Poisoning Severity Score (PSS) [23, 24], Sequential Organ Failure Assessment (SOFA) [25], Simplified Acute Physiology Score II (SAPS II) [26], and Practical Predicting Scoring System [4]. These scoring systems are complex and rely on subjective assessment of clinical information and laboratory investigations, which are difficult to acquire or accurately describe.

Several studies have assessed the role of vital status parameters in OP poisoning patient prognosis, though the findings are inconclusive to date [17, 27, 28]. One of the main reasons might be the conventional approach in many studies of using a single baseline measurement of vital status parameters to predict mortality. These parameters are dynamic and tend to change substantially and sometimes quite rapidly. In such a scenario, the approach of linking single measurements at the time of presentation with mortality during follow-up might not be an appropriate method. In such studies in which vital status parameters and laboratory investigations are observed at baseline only, an important question is whether the mean level of a parameter changes over time; if it does, it should be determined whether that change leads to certain latent groups that are different than the classifications made based on single baseline measurement of the same variable. Accordingly, this single observation approach might be prone to misclassification bias.

In general, latent class growth analysis (LCGA) provides a better alternative to observe and estimate growth trajectories over time for dynamic variables. Structural equation modelling (SEM) advances basic longitudinal analysis of data to include latent variable growth over time while modelling both individual and group changes using slopes and intercepts [29]. The traditional techniques employed are analysis of variance, multiple regression and multilevel models, which are variable-centred approaches; in contrast, LCGA is a person-centred approach focused on identifying unobserved subpopulations comprising similar individuals [30]. To the best of our knowledge, no previous study has assessed the latent trajectories of vital status parameters in OP poisoning patients and their relationship with mortality. Therefore, the aim of the present study was to analyse the growth trajectory of vital status parameters among OP poisoning patients using LCGA and determine relationships between classes of individuals based on individual response (vital parameters) patterns with mortality using survival analysis.

## Methods

This was a retrospective cohort of OP poisoning patients older than 13 years. All patients admitted from August 2010 to September 2016 at the medical intensive care unit (ICU) of a tertiary care hospital, Dr. Ruth K.M. Pfau/Civil Hospital Karachi, Pakistan, were included in this study. A total of 499 OP poisoning patients (of either sex) confirmed based on medical records were eligible during the six-month data collection period from June 2016 to November 2016. Dr. Ruth K. M Pfau/Civil Hospital Karachi is one of the largest tertiary care hospitals in the province of Sindh, Pakistan, with an annual patient turnover of approximately > 4,000,000.

### Study variables

Data for each patient were obtained from medical records, including demographics (age, sex), elapsed time since poison ingestion, and ICU stay. In addition to these parameters, vital status parameters (body temperature, systolic blood pressure, diastolic blood pressure, heart rate, respiratory rate and partial oxygen pressure) were obtained from medical records for an initial period of 2 days as per the completion of medical records. These vital status parameters were captured over time during the hospital stay as repeated measures in this study. In-hospital mortality data were recorded to assess the outcome of patients during the hospital stay.

### Latent classes

LCGA was used to detect trajectories of vitals over time. This falls under the umbrella of finite mixture modelling and is designed to detect latent classes over time of individuals following comparable movements of a determinant [31] . Our models used first- and second-order polynomials. Initially, data files were created for each of the vital status parameters and for each order. Per guidelines [32], models were prepared separately for each order, vital sign, and class (two/three/four). For every individual, we computed posterior probabilities for each trajectory by running all models. Latent classes extracted from the output files were created. Classes were later transformed in STATA with other demographic and vital status parameters. We approximated the trajectories by best-fitting based on the minimum Akaike Information Criterion (AIC), Bayesian Information Criterion (BIC) and high log likelihood [33]. These indices are suggestive of linear models compared to quadratic models for any class.

Our models used first- and second-order polynomials that best fit indices to find the maximum latent classes. The first-order polynomials exhibited a linear growth pattern when using data for 449 patients, which required at least three repeated observations. However, second-order polynomials revealed quadratic growth patterns required at least four repeated observations, with all four observations for only 424 patients. For all parameters based on fit indices, we found the best number of classes using first-order polynomials; an exception was temperature, for which we identified best classes using second-order polynomials.

### Statistical analysis

The data were entered and managed using Microsoft Office 365 Excel. Latent classes were generated from MPlus software, and inferential analysis and figures were plotted using STATA software version 15. Baseline differences among deceased and alive individuals were assessed. To detect differences in age, total ICU stay and vital status parameters between deceased and alive individuals, associations of mortality with sex were assessed using the Wilcoxon rank sum test based on data distribution.

For survival analysis, total ICU stay time was used to define risk time. We computed unadjusted and adjusted hazard ratios and respective confidence intervals for mortality by the assigned trajectory using the Cox proportional hazards models reported in Table 2. In multivariable analysis, we adjusted for age and approximate time elapsed since poison ingestion. We evaluated proportional hazards assumption by the phtest and smoothed Schoenfeld residuals plotted against time; the assumption was identified as satisfactory and not violated [34]. *P*-values < 0.05 were considered statistically significant.

### Ethical review

The study was approved by the Institutional Review Board of Dow University of Health Sciences (DUHS) (Ref No. IRB-560/DUHS/Approval/2015/75 dated 11th Jun 2015) and Board of Advanced Studies and Research (BASR) (BASR/No./02505/Sc. dated 2^nd^October 2015) of the University of Karachi-Pakistan.

## Results

Of 499 patients, records for 19 were excluded: 13 due to incomplete data; 2 due to incorrect registration; and 4 due to a self-reported history of chronic conditions such as hypertension, diabetes mellitus, osteoarthritis, asthma or pregnant women. Another 31 patients were excluded due to an ICU stay of less than 36 h, of whom 11 died. Thus, data for 449 patients, with a mean age of cohort 25.4 years (range 13–85 years), were included in the final analysis. The overall mortality was 13.4% (*n* = 60). Age and elapsed time since ingestion of poison were significantly higher among deceased compared to alive individuals, whereas sex, total ICU stay, and vital status parameters were not significantly different. (Table 1).

**Table 1:** Descriptive statistics (median (IQR)) of baseline characteristics with mortality

Basic Characteristics	Alive	Dead	***P***-value
**Linear Classes (3 OR 4 Time Points),** ***N*** **= 449**	389	60	
**Quadratic Classes (4 Time Points),** ***N*** **= 424**	370	54	
**Sex**			
Female (*N* = 236, %)	206 (87.3)	30 (12.7)	0.669^a^
Male (*N* = 213, %)	183 (85.9)	30 (14.1)	
**Age (years)**	22.0 (18.0–28.0)	28.0 (20.0–37.5)	0.0002^b^
**Elapsed Time (hours)**	4.4 (1.7–7.6)	8.5 (6.7–12.1)	< 0.001^b^
**ICU Stay (hours)**	111.0 (74.8–228.0)	164.6 (78.0–245.0)	0.445^b^
**Temperature (°F)**	98.5 (98.0–99.0)	98.5 (98.0–99.0)	0.333^b^
**SBP (mmHg)**	120.0 (100.0–130.0)	115.0 (100.0–140.0)	0.510^b^
**DBP (mmHg)**	70.0 (60.0–80.0)	70.0 (60.0–90.0)	0.366^b^
**Heart rate (minutes)**	110.0 (90.0–125.0)	111.0 (86.0–134.0)	0.496^b^
**Respiratory rate (minutes)**	22.0 (20.0–24.0)	20.0 (18.0–24.0)	0.360^b^
**PO**_**2**_ **(mmHg)**	99.0 (98.0–99.0)	99.0 (97.0–99.0)	0.097^b^

*IQR* interquartile range (25th–75th percentile), *PO*_*2*_ partial oxygen pressure, ^a^chi-square test, ^b^Wilcoxon rank sum test, *ICU* intensive care unit, *SBP* systolic blood pressure, *DBP* diastolic blood pressure

**Fig. 1:**
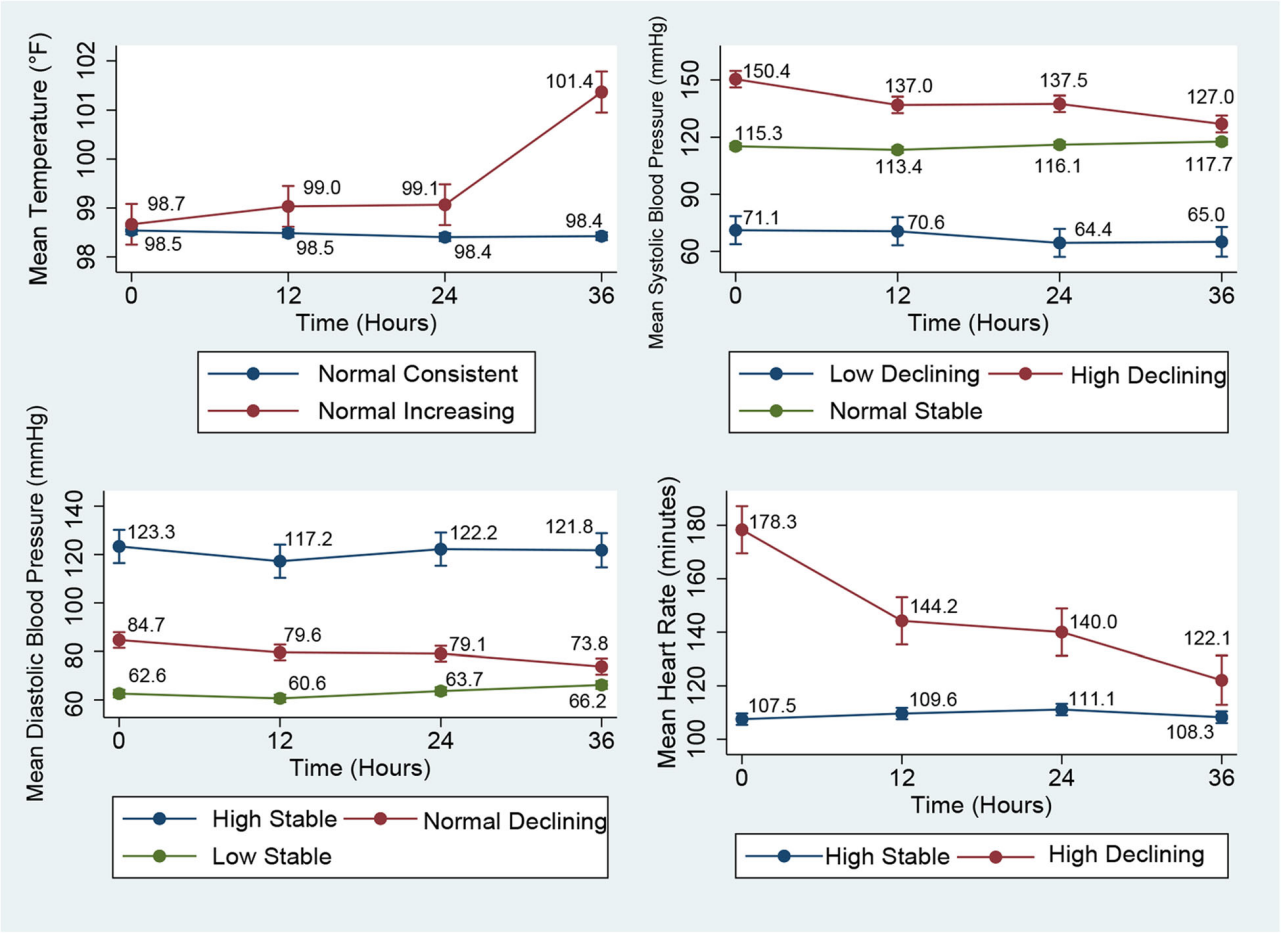
Trajectories of vital signs (temperature, SBP, DBP, heart rate)

**Fig. 2:**
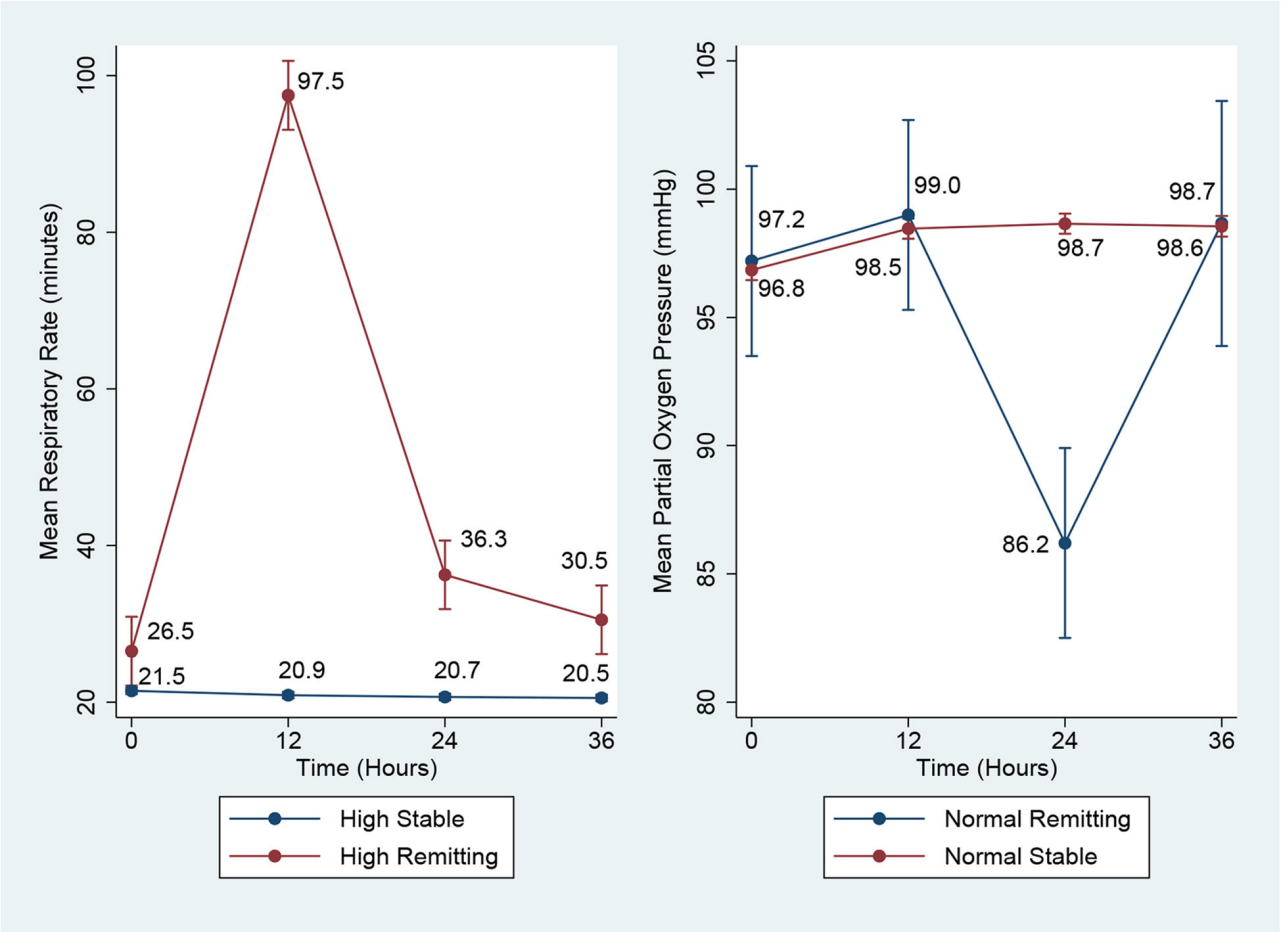
Trajectories of vital signs (respiratory rate, partial oxygen pressure)

We assigned labels to the trajectories based on their modelled graphic patterns (Figs. 1 and 2). We defined the first part of labels based on the start of vital sign ranges [35–39] (e.g., low, high, normal) and the second part on their repeated measure patterns (e.g., consistent, stable, declining, increasing, remitting (up then down or vice versa)). Word remitting refers to a pattern of multiple repetitions of low and high observations starting from low to high or vice versa. The “decreasing” designation used for other variables was applied for patients who had initially high values of a given variable, which then decreased during the course of observations; 36 h and “increasing” was used for patients who had initially low values, which then reduced the ICU stay by 36 h. Based on the mean intercept and slope of individual patterns, we identified two distinct trajectories (high-stable and high-declining) of heart rate in the 449 patients (mortality in high-stable was 50/424 = 11.8%; mortality in high-declining was 10/25 = 40.0%). (Fig. 1).

Three trajectories (high-declining, normal-stable, and low-declining) were identified for systolic blood pressure. Mortality for high-declining patients was 8/53 = 15.1%; it was 45/378 = 11.9% for normal-stable patients and 7/18 = 38.9% for low-declining patients. (Fig. 1).

Two trajectories (high-stable and high-remitting) were identified for respiratory rate. The mortality for the high-stable group was 58/445 = 15.1%, and that for the high-remitting group was 02/04 = 50.0%. (Fig. 2).

Two trajectories (normal-stable and normal-remitting) were also identified for partial oxygen pressure. The mortality for normal-stable patients and normal-remitting patients was 57/444 = 12.8% and 03/05 = 60.0%, respectively. (Fig. 2).

According to multivariable analysis after adjustment for age and elapsed time, patients with low-declining systolic blood pressure were three times more likely [HR: 3.0, 95% CI: 1.2–7.1, *P*-value = 0.014] to die compared with those who had a normal-stable systolic blood pressure and those in the high-declining heart rate trajectory were three times [HR: 3.0, 95% CI: 1.5–6.2, *P*-value = 0.002] more likely to die compared with those who had a high-stable heart rate. Patients in a high-remitting respiratory rate trajectory were six times [HR: 5.7, 95% CI: 1.3–23.8, *P-*value = 0.018] more likely to die compared with those who had a high-stable respiratory rate. Patients in the normal-remitting partial oxygen pressure trajectory were five times [HR: 4.7, 95% CI: 1.4–15.1, *P-*value = 0.010] more likely to die compared with those who had a normal-stable partial oxygen pressure. (Table 2).

**Table 2:** Relationship of mortality with vitamin status parameters = latent class growth analysis

Vital Parameters	Alive (***N*** = 389)	Dead (***N*** = 60)	Total	***P***-value	Unadjusted HR (95% C.I)	Adjusted HR (95% C.I)
**Temperature**	***N*** **= 370**	***N*** **= 54**				
Normal consistent	359 (87.8%)	50 (12.2%)	409	0.110^b^	1.0	1.0
Normal increasing	11 (73.3%)	4 (26.7%)	15		1.9 (0.7,5.5)	2.0 (0.7,5.7)
**SBP**						
Normal stable	333 (88.1%)	45 (11.9%)	378	0.004^a^	1.0	1.0
High declining	45 (84.9%)	8 (15.1%)	53		1.0 (0.5,2.1)	0.8 (0.3,1.7)
Low declining	11 (61.1%)	7 (38.9%)	18		3.0 (1.4,6.7)	3.0 (1.2,7.1)
**DBP**						
Normal declining	68 (82.9%)	14 (17.1%)	82	0.256^a^	1.0	1.0
High stable	14 (77.8%)	4 (22.2%)	18		1.3 (0.4,3.9)	1.5 (0.5,4.8)
Low stable	307 (88.0%)	42 (12.3%)	349		0.8 (0.5, 1.5)	1.0 (0.5, 1.8)
**Heart Rate**						
High stable	374 (88.2%)	50 (11.8%)	424	0.001^b^	1.0	1.0
High declining	15 (60.0%)	10 (40.0%)	25		2.7 (1.4,5.4)	3.0 (1.5,6.2)
**Respiratory Rate**						
High stable	387 (87.0%)	58 (13.0%)	445	0.088^b^	1.0	1.0
High remitting	2 (50.0%)	2 (50.0%)	4		3.8 (0.9,15.5)	5.7 (1.3,23.8)
**PO**_**2**_						
Normal Stable	387 (87.2%)	57 (12.8%)	444		1.0	1.0
Normal Remitting	2 (40.0%)	3 (60.0%)	5	0.019^b^	4.9 (1.5,15.9)	4.7 (1.4,15.1)

Adjusted covariate: age & elapsed time; ^a^Chi-square test; ^b^Fisher exact test; *SBP* systolic blood pressure, *DBP* diastolic blood pressure, *PO*_*2*_ partial oxygen pressure, *HR* hazard ratio

## Discussion

### Summary of findings

Among OP poisoning cases, older age and longer elapsed time since ingestion were significantly associated with mortality. Furthermore, individuals in the low-declining systolic blood pressure trajectory and high-declining heart rate trajectory had significantly higher mortality than those in the normal-stable systolic blood pressure trajectory and the high-stable heart trajectory. Similarly, patients in the high-remitting respiratory rate trajectory and those in the normal-remitting partial oxygen pressure trajectory had a nearly sixfold and fivefold increased risk of mortality, respectively, compared with those who were in the high-stable respiratory rate and normal-stable partial oxygen pressure categories. These findings suggest that routine clinical parameters, if observed repeatedly, might be useful clinical tools to identify high-risk groups of patients who might experience considerably high mortality. The mortality in our study is comparable to previously published papers on OP poisoning from different regions, including neighbouring countries [4, 9, 18, 27, 40–42]; however, our study appeared to show higher (13.4%) mortality compared to a published paper (4.11%) [42] from another tertiary care setting in our metropolitan city. Although the average ages of patients in their study and our study were comparable, a significantly higher percentage of women (73%) was enrolled in the previously published study [42] than in our study (52.5%). Therefore, the low mortality in the previous study might be explained by a high percentage of women because it is well known now that the risk of mortality and success of suicidal attempts due to poisoning among females tends to be lower than that of men [43–46]. Second, in the previous study, patients were kept in the general medical ward for treatment, suggesting that the poisoning cases were mild to moderate. However, in our cohort, the patients all has been directly transferred from emergency to intensive care units, which reflects predominantly more severe forms of poisoning cases with deteriorating clinical condition and explains the high mortality in our study compared to the previous study. Older age and longer time elapsed since ingestion of poison were significant predictors of mortality in our series, and the findings are quite consistent with previously published studies [18, 27, 42].

Trajectory analysis showed low-declining systolic blood pressure and high-declining heart rate to be significantly associated with an increased risk of mortality among OP poisoning patients. The result for SBP was in contrast with multiple studies showing that it was not associated with mortality [26, 27, 42] . The heart rate result was somewhat supported by European and Asian studies, which reported that a high heart rate is associated with mortality [27, 42, 47]; nonethelesss, it was not linked with mortality in two Asian studies [48, 49] . Trajectories of body temperature and diastolic blood pressure did not show any significant relationship with mortality. LCGA in our study indicated that the high-remitting trajectory of respiratory rate was linked to mortality. However, these results were in contrast with previous studies, as high temperature and low respiratory rate have exhibited significance with mortality in multiple studies [26, 47, 49, 50], though nonsignificance for respiratory rate [47] and temperature [48] was observed in a study of OP poisoning patients. Multiple studies have investigated vital signs but not PO_2_ [47, 50, 51], whereas our study showed that a normal-remitting partial oxygen pressure trajectory is linked to an increased risk of mortality.

Vital status parameters and their relationship with mortality among OP poisoning patients were not entirely clear in previous studies [19, 42, 52]. One reported that OP poisoning patients had a normal temperature at the time of admission but that their mean tympanic temperature increased considerably after atropine administration during the hospital stay until 72 h after admission [53] . Moreover, previous studies only included baseline measurements of vital status parameters and examined their relationship with mortality. Our study is unique in that it accounted for multiple observations of vital status parameters in the first 36 h after poisoning and linked them to mortality. Our approach is also closer to real life scenarios, in which such OP poisoning patients display varying levels of these parameters; therefore, our findings may be more applicable to clinical settings.

Commonly used scoring systems heavily depend on clinical and laboratory investigational information [4, 20–26] Furthermore, most scoring systems rely on single measurements, usually at the time of patient admission. The LCGA used in this study is a person-centred approach in which latent classes identify trajectories of patients based on repeated measures of very routine clinical parameters [54] . Individuals are similar within classes but differ across them [30]. Trajectories of routine clinical parameters are much more useful and handier for clinical utility and decision making than other lab-based investigations used in different scoring systems.

### Strength and weakness

This study has several strengths and limitations, which should be considered when drawing any conclusions. This study was unique because multiple observations of vital status parameters in the first 36 h of admission into intensive care were made and its relationship with mortality among OP poisoning patients was determined. To the best of our knowledge, this is the first study to assess the relationship of vital status parameters with mortality using latent class growth analysis. The current study had a reasonable sample size by including data from several years from the intensive care unit of a tertiary care hospital, which provides generalizable results in our context.

In terms of weaknesses, the amount of poison ingested was obtained from medical records, which might not have been correctly estimated by the clinicians. There is some evidence that suggests that the difference in the type of insecticide ingested can influence the outcome [23, 55] . As in the majority of the cases in our cohort, we could not ascertain the type of poison ingested, which might also have some influence on mortality between different trajectories. This remains a limitation of our study. Another important point is the retrospective design of the study, which might have compromised the quality of the data to some extent. Nevertheless, our retrospective study provides the first application of trajectory analysis to predicting mortality among OP poisoning patients. Established biomarkers such as anticholinesterases, malondialdehyde, and glutathione were not included in our analysis because the repeated measures of these markers were not available in medical records for trajectory analysis. However, this study examined accessible, noninvasive, inexpensive vital status parameters that have more clinical utility and are much more convenient than laboratory-based markers.

## Conclusion

Latent classes of systolic blood pressure, heart rate, respiratory rate and partial oxygen pressure were significant predictors of mortality among acute OP poisoning patients. Large-scale studies with the addition of biomarkers are needed to determine the clinical utility of these trajectories of vital status parameters in determining the prognosis of patients with acute OP poisoning.

## Data Availability

Data included in the current study are not publicly available to ensure confidentiality of the patients but are available from the corresponding author on reasonable request.

## Abbreviations

OP: Organophosphorus
LCGA: Latent class growth analysis
ICU: Intensive care unit
GCS: Glasgow Coma Scale
AIC: Akaike information criteria
BIC: Bayesian information criteria
HR: Hazard ratio
SBP: Systolic blood pressure
DBP: Diastolic blood pressure
SEM: Structural equation models
MV: Mechanical ventilation
